# Fatty Acid Composition and Volatile Profile of *M. longissimus thoracis* from Commercial Lambs Reared in Different Forage Systems

**DOI:** 10.3390/foods9121885

**Published:** 2020-12-17

**Authors:** Yangfan Ye, Graham T. Eyres, Mariza G. Reis, Nicola M. Schreurs, Patrick Silcock, Michael P. Agnew, Patricia L. Johnson, Paul Maclean, Carolina E. Realini

**Affiliations:** 1 AgResearch Grasslands, Tennent Drive, Palmerston North 4442, New Zealand; Yangfan.Ye@agresearch.co.nz (Y.Y.); Mariza.GomesReis@agresearch.co.nz (M.G.R.); michael.agnew@agresearch.co.nz (M.P.A.); Paul.Maclean@agresearch.co.nz (P.M.); 2 Animal Science, School of Agriculture and Environment, Massey University, Private Bag 11222, Palmerston North 4442, New Zealand; N.M.Schreurs@massey.ac.nz; 3 Department of Food Science, University of Otago, P.O. Box 56, Dunedin 9054, New Zealand; graham.eyres@otago.ac.nz (G.T.E.); pat.silcock@otago.ac.nz (P.S.); 4 AgResearch Invermay, Puddle Alley, Mosgiel 9092, New Zealand; Tricia.Johnson@agresearch.co.nz

**Keywords:** meat, lipids, volatiles, nutrition, production system

## Abstract

Animal production factors can affect the fatty acid and volatile profile of lamb meat. The fatty acid and volatile composition of the *M. longissimus thoracis* was evaluated from 150 lambs from 10 groups of commercial lambs that differed in age, sex, diet and breed, from three farms, which represent typical forage lamb production systems in New Zealand. The meat from 4-month-old composite lambs slaughtered at weaning had a similar polyunsaturated to saturated fatty acid ratio compared to 6- to 8-month-old composite lambs, but a greater ratio than that of 12-month-old Merino lambs (*p* < 0.05), with all ratios being lower than the recommended ≥0.45. All lamb production systems produced meat with an omega-6 to omega-3 ratio below 1.5, well below the recommended ratio ≤ 4.0. Meat from 4-month-old lambs had higher C12:0, C14:0 and C16:0 and lower C18:0, reflecting the composition of the milk diet, resulting in higher atherogenic index than meat from other animal groups, while meat from 12-month-old Merino lambs, with lower content of polyunsaturated fatty acids, showed higher thrombogenic index. Meat from lambs processed at weaning contained the greatest concentration of eicosapentaenoic and docosahexaenoic acids, which would qualify as a ‘source’ or ‘good source’ of these target fatty acids based on the Commission of Regulation of the European Union or the Food Standards Australia New Zealand guidelines, respectively. Volatiles were extracted from the headspace of raw lean meat and 36 volatile compounds were identified. The abundance of carbon disulphide, isododecane, heptanal, 2,5-hexanediol and 3-octanone and pentanoic, octanoic, nonanoic and heptanoic acids was similar between all groups of lambs. Meat from 12-month-old Merino lambs had low abundance of acetic, propanoic, butanoic and hexanoic acids, and hexanal, octanal and dimethyl sulphide. For 6- to 8-month-old composite lambs, hexanal, octanal and nonanal were present at higher relative abundance in meat from lambs that grazed on chicory than perennial ryegrass. The significant differences in the fatty acid and volatile profiles in meat from 12-month-old Merino lambs compared with lambs slaughtered at weaning or further grazed on red clover, chicory or mixed pasture may result in distinctive nutritional value and lamb flavour.

## 1. Introduction

Red meat is an important source of protein, fatty acids and vitamins, which are essential for human health [1]. Dietary fat from meat plays an important role in health maintenance and disease prevention, delivers nutrients such as vitamins A, D, E, carotenoids [2], long-chain omega-3 polyunsaturated fatty acids (LC-n-3-PUFA) and conjugated linoleic acid (CLA), the latter of which is almost exclusively sourced from red meat and dairy products [3]. A high n-3 fatty acids intake diet has been reported to decrease the risk of coronary heart disease [4], while a lack of n-3 fatty acids intake in childhood is linked to behavioural and learning disorders such as attention deficit hyperactivity disorder [5]. Conversely, a high intake of saturated fatty acids (SFA) and *trans*-fatty acids adversely affects glucose metabolism and insulin resistance, which have been associated with Type II diabetes and other health concerns such as cardiovascular disease [6]. The U.S. Department of Agriculture and Human Health Services [7] has verified that the cholesterol-raising fatty acids include SFA from C12:0 to C16:0, which are commonly found in meat from ruminant animals [8].

An increasing awareness by consumers on how food influences their health [9] poses challenges for lamb producers and processors, where consumers look for food items with a higher concentration of n-3 fatty acids and lower amounts of SFA. In Australia and New Zealand, food containing over 22 and 44 mg per 100 g serve of eicosapentaenoic acid (EPA) plus docosahexaenoic acid (DHA) may be considered as a ‘source’ and a ‘good source’ of them [10], while over 40 and 80 mg per 100 g serve of EPA plus DHA are needed for a food to be considered as a ‘source’ and a ‘good source’ of them in the European Union [11].

Understanding the effect of diverse forage production systems on lamb fatty acid composition, in particular n-3 fatty acids, would provide an opportunity to differentiate lamb products for discerning markets. Production systems can vary depending on animal characteristics including breed, sex and age at slaughter, and management factors such as animal diet, which are all factors that have been noted to contribute to variations in the fatty acid composition of meat [8]. For instance, the fatty acid profile of lamb becomes more saturated as the animal becomes older [12]. Female lambs have a tendency to deposit more fat and provide more saturated fatty acid profile than male lambs at a given weight [13,14]. Thus, there is potential for the diversity in lamb production systems between countries and between farms within countries to contribute to differences in the fatty acid composition of meat.

Forage-based animal diets enhance the proportion of n-3-PUFA in meat, and often enrich meat with antioxidants compared to the meat from animals fed concentrate-based diets [15]. However, there is limited information regarding the fatty acid composition of meat from lambs finished in different grazing systems. In turn, the oxidation derivatives of PUFA contribute to the pastoral flavour in meat from ruminant animals [16]. Lipid oxidation in meat occurs in phases, where the primary phase involves the removal of hydrogen from a methylene group, which forms a radical that can be rearranged, and the secondary phase involves the decomposition of hydroperoxides and leads to the formation of volatile compounds, including some aldehydes, alcohols and ketones [17]. Although many volatile compounds responsible for meat flavour arise from heat-induced reactions during cooking, secondary oxidation compounds present in the raw meat may remain after cooking and adversely or positively affect flavour perception [18].

The broad range of forage-based commercial production systems utilised in New Zealand suggests that there is potential for variation in fatty acid and volatile profiles in lamb meat. The objective of this study was to evaluate the meat fatty acid and volatile profiles of lambs from diverse forage-based production systems in New Zealand and to compare the fatty acid levels in meat with published guidelines for human consumption.

## 2. Materials and Methods

### 2.1. Animals and Management

To encompass a range of forage-based production systems, lambs were sourced from three commercial farms north of Invercargill, New Zealand (Table 1), following regular animal handling farm practices. Thus, animal ethics approval was not needed for this study under current New Zealand regulations. These three farms provided ten production systems that were selected based on discussions among stakeholders and meat scientists to represent typical forage production systems and lamb meat commonly exported from New Zealand to key international markets. The study was not focused on the evaluation of the individual impact of animal sex, diet, genetics and their interactions on meat composition, but on the evaluation of the range of fatty acids and volatiles in lamb meat from ten different representative and industry-relevant production systems. The ten production systems included: 4-month-old wether lambs of a composite breed at weaning (WEAN-W-4), 6- to 8-month-old wether and ewe lambs of a composite breed that had been grazing perennial ryegrass-based pasture (GRASS-W-6-8 and GRASS-E-6-8, respectively), 6- to 8-month-old wether and ewe lambs of a composite breed that had been grazing chicory (CHIC-W-6-8 and CHIC-E-6-8, respectively), 6- to 8-month-old wether and ewe lambs of a composite breed that had been grazing red clover (REDC-W-6-8 and REDC-E-6-8, respectively), 6- to 8-month-old wether lambs of composite breed that had been grazing a mixture of perennial ryegrass, red- and white-clover mixed pasture (MIX-W-6-8) and 12-month-old wether and cryptorchid Merino lambs that had been grazing a mixed pasture (PMER-W-12 and PMER-C-12, respectively). Fifteen lambs from each production system were randomly selected and identified at slaughter from a larger group of lambs.

### 2.2. Slaughter and Sampling

Lambs were processed at Alliance Group Ltd., Lorneville plant, Invercargill, New Zealand. The 4- and 12-month-old lambs were slaughtered in December 2017 and the 6- to 8-month-old lambs were slaughtered in March 2018. All lambs were electrically stunned, exsanguinated and dressed according to standard commercial procedures. The *Longissimus thoracis* (loin) muscle was removed from the carcass, vacuum-packed and chilled at −1.5 °C for 21 days, then frozen at −20 °C until further analysis. A detailed description of animal groups, sample collection and carcass and meat quality characteristics corresponding to this study was previously reported in Reference [19].

### 2.3. Fatty Acid Composition

Prior to fatty acid composition analysis, all samples were freeze-dried (Cuddon Freeze Drier, Blenheim, New Zealand) and ground into a fine powder. Fatty acid concentrations were measured using trans-methylation of the fatty acids and quantification by gas chromatography (GC), as described by Agnew et al. [20]. Freeze-dried ground meat (300 mg), 4 mL of toluene, 0.3 mL of internal standard (C11 triglyceride in toluene) and 4 mL of 5% of sulphuric acid in methanol were mixed thoroughly by vortex and incubated at 70 °C for 2 h, with mixing every 30 min during the incubation time. After 25 min of equilibration at room temperature, 5 mL of saturated NaCl was added, mixed and then centrifuged at 2300 rpm for 2 min to separate solvent layers. The top layer containing the fatty acid methyl esters (FAME) was transferred into a 1.5 mL GC autosampler vial (Hewlett Packard, model 6890). The GC was a Shimadzu GC-2010 plus (Shimadzu Corporation, Kyoto, Japan) with a flame ionisation detector (FID). The column was a Restek RTX 2330 column of 105 m length, 0.25 mm i.d., and 0.20 μm film thickness (Restek Corporation, Bellefonte, PA, USA). The thermal program used an initial temperature of 175 °C for 17 min, which was increased to 220 °C at a rate of 6 °C per min and held for 10 min. The carrier gas was hydrogen with a linear velocity of 50 cm/s. The injection volume was 1 μL, with a split ratio of 80:1. The injector temperature was 260 °C and the detector temperature was 300 °C.

The peak areas obtained from the GC were integrated using the Shimadzu Lab-solution software (version 4.20, Shimadzu, Kyoto, Japan) in a post-run analysis. Peaks were identified by comparison of their retention times with those of commercial standard mixtures (FAME mix 37 components from Supelco Inc., Bellefont, PA, USA) and quantified by using the internal standard (C11:0) and theoretical FID response factors [20]. The equations for generating the response and conversion factors to quantify individual fatty acids from FAME were obtained from American Oil Chemists’ Society 6th edition (AOCS Ce1f-96, Ce 1 h-05 and Ce 1i-07). Individual fatty acid content was expressed as mg per 100 g of raw meat (*M. longissmus thoracis*) and as a percentage of total fatty acids. For 10% of the samples, a randomly allocated duplicate sample was analysed to verify consistency. Major fatty acid groups and PUFA:SFA ratio, n-6:n-3 ratio, thrombogenic index [21], atherogenic index [21], hypocholesterolaemic/Hyperdolesterolaemic (h/H) ratio [22] and nutritional ratio [23] were calculated from the relevant individual fatty acids to enable comparison with human nutritional guidelines.

### 2.4. Volatile Compounds Analysis

The samples for volatile analysis were analysed in daily batches with 10 samples in each batch. One loin from each production system was randomly chosen for each daily batch. From each loin, duplicate samples of 4 g of frozen lean meat were removed as 5 mm cores and placed in the bottom of 20 mL solid-phase microextraction (SPME) vials. Samples were kept at 4 °C overnight until analysis. Internal standard (50 μL, 1.25 mg/L fenchol in water, 99 % purity, Sigma-Aldrich, Madrid, Spain) was added to a 250 µL insert vial sitting next to the meat within the SPME vials. To minimise the chance of volatile compound changes, samples were held in the auto sampler tray for no longer than 2.5 h. To minimise analysis sequence effects, samples were analysed using a balanced random order, and blanks (empty vials) were also run every day to check background signals.

The vial was equilibrated at 37 °C for 5 min in the automated sample preparation unit. Following equilibration, a 50/30 μm Divinylbenzene-Carboxen-Polydimethylsiloxane (DVB/CAR/PDMS) fibre of the auto sampler (PAL RSI 85, Switzerland) was exposed to the headspace of the unstirred sample for 30 min at 37 °C and then desorbed directly in the injection port of a 6890N gas chromatograph coupled to a 5975B VL mass spectrometric detection system (Agilent Technologies, Inc., Santa Clara, CA, USA). Desorption occurred for 5 min at 240 °C, and 2 min in splitless mode followed by 3 min with a purge flow of 60 mL/min. Helium was the carrier gas at a flow rate of 1.2 mL/min. A ZB-WAX capillary column (Phenomenex, Torrance, CA, USA) of 60 m × 0.32 mm i.d. × 0.5 μm film thickness was used for the separation. The oven temperature was initially 50 °C for 2 min, then raised by 10 °C/min to 240 °C and held at this temperature for 10 min. After desorption, the fibre was cleaned for 2 min at 270 °C. For mass spectrometry, the transfer line temperature was 230 °C and the trap temperature was 150 °C with an emission current of 34.61 μA. The global run time was recorded in full scan mode (*m*/*z* 29–300 mass range) and the chromatographic data were analysed by MSD Chemstation (version F.01.012317, Agilent Technologies, Inc., Santa Clara, CA, USA).

Compound identification was carried out by spectra comparison using the Wiley7Nist05 Library (Wiley & Sons Inc., Weinheim, Germany) supported by linear retention indexes (LRI) relative to a series of alkanes (C7–C26) compared to 11th edition National Institutes of Standards and Technology Mass Spectral Library. Due to some selected analytes coeluting with other compounds, the selected masses were used for integration. The integrated areas of the selected compound ions were divided by the area of the internal standard ion (*m/z* 81) to give relative abundances.

### 2.5. Statistical Analysis

Permutation analysis of variance (permANOVA) was performed using the lmPerm package version 2.1.0 [24] in R version 3.0.2 [25], that included the production system as a fixed effect. In addition, pairwise comparisons were made between ewe, wether and cryptorchid lambs fed the same diet within a farm (Farm A: REDC-W-6-8 vs. REDC-E-6-8, Farm B: PMER-W-12 vs. PMER-C-12, Farm C: GRASS-W-6-8 vs. GRASS-E-6-8 and CHIC-W-6-8 vs. CHIC-E-6-8), and between lambs that grazed chicory and perennial ryegrass (Farm A: CHIC-W-6-8 vs. GRASS-W-6-8 and CHIC-E-6-8 vs. GRASS-E-6-8). These combined factors were grouped according to their false discovery rate corrected [26] *p*-values and were considered significant at a probability level of <0.05.

In order to summarise the relative differences amongst samples in relation to their overall fatty acid and volatile profiles, a Principal Component Analysis (PCA) was performed on the content of identified fatty acids (mg/100 g raw meat) and relative abundance of identified volatiles using the mixOmics package version 6.12.2 [27] and plotted using the factoextra package version 1.0.7 [28] in R.

## 3. Results

### 3.1. Quantitative Fatty Acids

**Figure 1:**
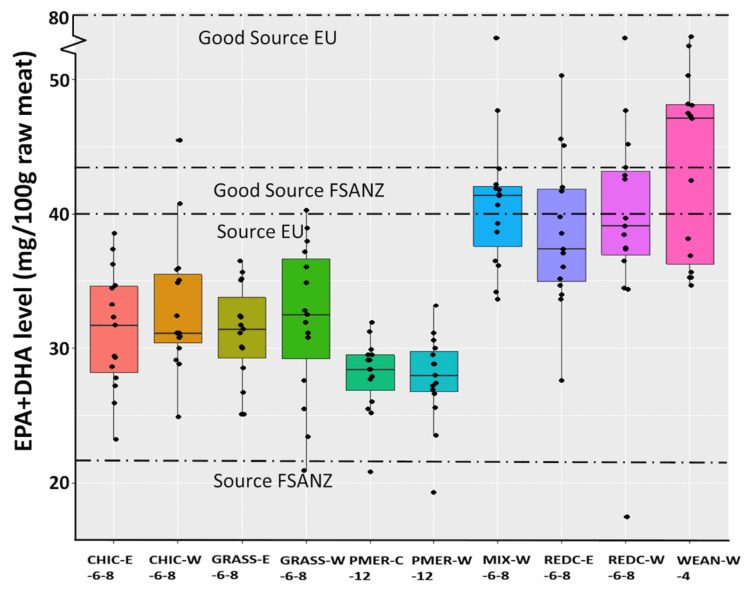
Box plot of EPA + DHA content in *Longissimus thoracis* for each lamb group. Horizontal dashed lines indicate minimum values for meat to be considered as a ‘source’ of EPA + DHA (22 mg/100 g muscle) or a ‘good source’ (44 mg/100 g muscle) according to Food Standards of Australia and New Zealand (FSANZ), and minimum values to be considered as a ‘source’ (40 mg/100 g muscle) or a ‘good source’ (80 mg/100 g muscle) of EPA + DHA fatty acids according to the Commission Regulation of European Union (EU).

The total fatty acid content and SFA, monounsaturated fatty acids (MUFA) and branched-chain fatty acids (BCFA) content were greater in PMER-W-12 lambs than in 6–8-month-old lambs that grazed perennial ryegrass, chicory or red clover (*p* < 0.05; Table 2). The SFA and BCFA content were greater in WEAN-W-4 lambs than in lambs that grazed on chicory, and the PUFA content was greater in WEAN-W-4 lambs than in other lambs slaughtered at 6- to 12-months old. Highest content of n-6 (185 mg/100 g) and n-3 (159 mg/100 g) fatty acids were observed in WEAN-W (Table 2; *p* < 0.05). The long-chain n-3 PUFA content was greater in WEAN-W-4 lambs compared to GRASS-W-6-8, GRASS-E-6-8, CHIC-W-6-8, CHIC-E-6-8, REDC-E-6-8, PMER-W-12 and PMER-C-12 lambs, with REDC-W-6-8 and MIX-W-6-8 lambs as intermediate groups. The combined EPA (20:5n-3) plus DHA (22:6n-3) content was greater in WEAN-W-4, REDC-W-6-8, REDC-E-6-8 and MIX-W-6-8 lambs than from GRASS-W-6-8, GRASS-E-6-8, CHIC-E-6-8, PMER-W-12 and PMER-C-12 lambs, and the CHIC-W-6-8 lambs were an intermediate group (Table 2, Figure 1).

When comparing within the 6- to 8-month-old lambs, the total fatty acid and SFA content was similar, but the MUFA and BCFA content was greater in MIX-W-6-8 lambs than in CHIC-E-6-8 lambs, and the PUFA content was greater in REDC-W-6-8 lambs than in GRASS-W-6-8 and GRASS-E-6-8 lambs (*p* < 0.05; Table 2), while the CHIC-W-6-8, CHIC-E-6-8, REDC-E-6-8 and MIX-W-6-8 lambs were intermediate groups.

### 3.2. Fatty Acid as a Proportion of Total Fatty Acids

The proportion of SFA was greater in Merino lambs compared with that of lambs (wethers and ewes) that grazed on chicory, red clover or mixed pasture (*p* < 0.05; Table 3), and WEAN-W-4 lambs was an intermediate group. The WEAN-W-4 lambs had the greatest proportion of C16:0 and lowest proportion of C18:0, while GRASS-W-6-8 lambs had the lowest proportion of C16:0 and PMER-W-12 had the greatest proportion of C18:0. The 12-month-old Merino lambs had a greater (*p* < 0.05; Table 3) proportion of BCFA than the CHIC-W-6-8, CHIC-E-6-8 and REDC-E-6-8 lambs.

The proportions of iso-C17:0 and anteiso-C17:0 were greatest in PMER-C-12 and PMER-W-12 lambs, respectively. Lowest and highest C18:1 *trans*-9 and MUFA proportions were observed in WEAN-W-4 lambs and Merino lambs respectively, among the production systems considered. For lambs at 6 to 8 months old, the REDC-W-6-8, CHIC-W-6-8 and CHIC-E-6-8 lambs had a lower proportion of C18:1 *cis*-9 and MUFA compared to MIX-W-6-8 lambs (*p* < 0.05; Table 3). The proportion of PUFA was lower (*p* < 0.05; Table 3) in Merino lambs compared to lambs slaughtered at 4 to 8 months old. The proportion of PUFA was greatest in lambs grazed on chicory but did not differ from lambs grazed on red clover or perennial ryegrass (*p* > 0.05, Table 3). C18:2n-6 and C18:3n-3 formed the highest proportion of PUFA in all lambs, and the PMER-W-12 lambs had the lowest proportion of C18:2n-6 and C18:3n-3 among all production systems considered. The proportion of conjugated linoleic acid isomer C18:2 *cis*-9, *trans*-11 was greatest in WEAN-W-4 lambs (1.36%) and lowest in PMER-C-12 lambs (0.74%), all the 6- to 8-month-old lambs were intermediate groups (0.90–1.08%) and did not differ from each other. The proportion of EPA plus DHA was lower in PMER-W lambs than lambs that slaughtered at 4 to 8 months old (*p* > 0.05, Table 3) and no difference was observed between WEAN-W-4 lambs and lambs slaughtered at 6 to 8 months old.

### 3.3. Nutrition Indexes

A lower PUFA:SFA ratio was observed in Merino lambs than in lambs slaughtered at 4 to 8 months old (*p* < 0.05; Table 4), and the greatest PUFA:SFA ratio was observed in lambs grazed on chicory. The n-6:n-3 ratio was highest in CHIC-E-6-8 and lowest in MIX-W-6-8 lambs, which also differed from GRASS-W-6-8 and CHIC-W-6-8. The atherogenic index value and the nutritional ratio were greater, while the h/H ratio was lower in WEAN-W-4 lambs compared with lambs from the other production systems (*p* < 0.05; Table 4), and the thrombogenic index value was greater in the 12-month-old Merino sheep than in the other production systems (*p* < 0.05; Table 4).

### 3.4. Volatiles from Raw Meat

#### 3.4.1. Acids

The top three most abundant volatile acids were acetic acid, butanoic and hexanoic acid. Acetic acid was relatively less abundant in the headspace of PMER-C-12 lambs compared to WEAN-W-4, GRASS-E-6-8, CHIC-W-6-8, CHIC-E-6-8, REDC-W-6-8 and REDC-E-6-8 (*p* < 0.05; Table 5). Butanoic acid was less abundant in 12-month-old Merino lambs than WEAN-W-4, GRASS-W-6-8, CHIC-E-6-8, REDC-W-6-8, REDC-E-6-8 and MIX-W-6-8 lambs (*p* < 0.05; Table 5). Also, hexanoic acid was less abundant in 12-month-old Merino lambs compared to 6- to 8-month-old lambs grazing chicory (CHIC-W-6-8, CHIC-E-6-8) or a mixed pasture (MIX-W-6-8; *p* < 0.05; Table 5).

#### 3.4.2. Alcohols

More abundant 1-pentanol was observed in CHIC-W-6-8, REDC-W-6-8 and MIX-W-6-8 than WEAN-W-4, GRASS-W-6-8, GRASS-E-6-8, PMER-W-12 and PMER-C-12 (*p* < 0.05; Table 5). 1-penten-3-ol was relatively more abundant in the headspace of CHIC-W-6-8, REDC-W-6-8 and MIX-W-6-8 lambs compared to WEAN-W-4, GRASS-W-6-8, GRASS-E-6-8, PMER-W-12 and PMER-C-12 (*p* < 0.05; Table 5). 1-octen-3-ol was more abundant in CHIC-W-6-8 and MIX-W-6-8 lambs compared to WEAN-W-4, GRASS-W-6-8, GRASS-E-6-8, REDC-E-6-8, PMER-W-12 and PMER-C-12 (*p* < 0.05; Table 5).

#### 3.4.3. Aldehydes

2-Methylbutanal was more abundant from the WEAN-W-4, REDC-W-6-8, REDC-E-6-8, MIX-W-6-8 and PMER-W-12 lambs than 6- to 8-month-old lambs that had grazed a chicory diet (*p* < 0.05; Table 5). The relative abundance of hexanal was greater in CHIC-W-6-8, CHIC-E-6-8, REDC-W-6-8 and MIX-W-6-8 lambs than the GRASS-W-6-8, GRASS-E-6-8, PMER-W-12 and PMER-C-12 lambs (*p* < 0.05; Table 5). The relative abundance of nonanal was greater in CHIC-W-6-8, REDC-W-6-8 and MIX-W-6-8 lambs compared to WEAN-W-4, GRASS-W-6-8, GRASS-E-6-8, PMER-W-12 and PMER-C-12 lambs (*p* < 0.05; Table 5).

#### 3.4.4. Ketones

Acetone was less abundant in the headspace of chicory-fed lambs slaughtered at 6–8 months of age (CHIC-W-6-8, CHIC-E-6-8) compared to WEAN-W-4, GRASS-W-6-8, REDC-W-6-8, REDC-E-6-8, MIX-W-6-8 and PMER-C-12 lambs (*p* < 0.05; Table 5). Within the 6- to 8-month-old group of lambs, a greater abundance of acetoin was found in chicory-fed lambs and red clover-fed ewe lambs than lambs fed a mixed pasture (*p* < 0.05, Table 5). The abundance of 2-butanone and 2-heptanone in the headspace was lower in CHIC-W-6-8, CHIC-E-6-8 and GRASS-E-6-8 lambs compared to WEAN-W-4, REDC-W-6-8, REDC-E-6-8 and MIX-W-6-8 lambs (*p* < 0.05; Table 5).

#### 3.4.5. Hydrocarbons and Furans

Beta-pinene was present at a greater abundance in GRASS-E-6-8, RED-C-6-8, REDC-E-6-8 and MIX-W-6-8 lambs compared to WEAN-W-4 lambs (*p* < 0.05; Table 5). PMER-C-12 lambs had the greatest abundance of Z-2-octene. The only furan detected, 2-ethylfuran, was more abundant from GRASS-E-6-8, CHIC-W-6-8 and MIX-W-6-8 lambs compared to WEAN-W-4, GRASS-W-6-8, PMER-W-12 and PMER-C-12 lambs (*p* < 0.05; Table 5).

#### 3.4.6. Sulphur Compounds

Although carbon disulphide was one of the greatest detected compounds in the headspace in the current study, its abundance in lambs in different production systems was similar (*p* > 0.05; Table 5). A greater relative abundance of dimethyl sulphide was found in ewe lambs that grazed chicory and wether lambs that grazed mixed pasture compared to lambs weaned and slaughtered at 4 months of age, ewe and wether lambs that had grazed perennial ryegrass and 12-month-old Merino lambs (*p* < 0.05; Table 5). The abundance of dimethyl sulfone was greater in lambs that had grazed red clover and for 12-month-old Merino lambs compared to GRASS-W-6-8 and CHIC-E-6-8 lambs (*p* < 0.05, Table 5).

### 3.5. Principal Component Analysis

**Figure 2:**
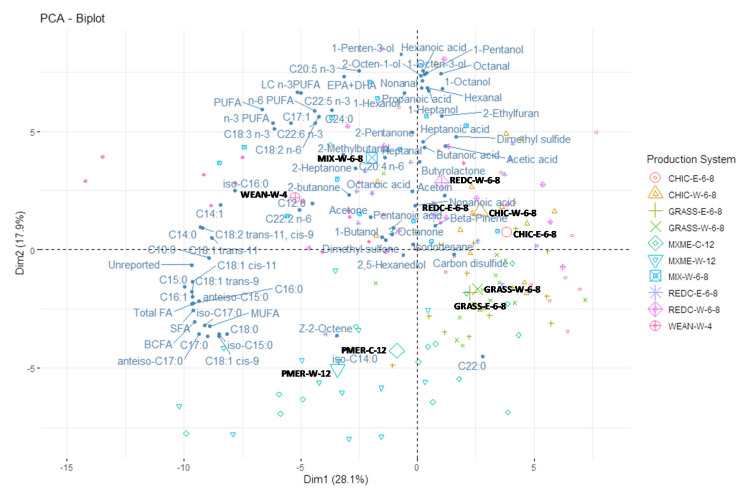
Principle component analysis (PCA) biplot of fatty acids content (mg/100 g) and volatile compounds identified in raw lamb meat (*Longissimus thoracis*) as affected by lamb production system.

To visualise differences in fatty acid content (mg/100 g) and volatile profiles among the different lamb groups, a PCA biplot is presented in Figure 2. The first dimension (Dim1) of PCA explained 27.1% of the total variation in fatty acid and volatile composition, and the second dimension (Dim2) explained 18.2% of the total variation. Dim1 separated the animal groups based on their total fatty acid content, where MIX-W-6-8, WEAN-W-4, PMER-W-12 and PMER-C-12 are positioned on the left side of the plot, opposite to REDC-W-6-8, REDC-E-6-8, CHIC-W-6-8, CHIC-E-6-8, GRASS-W-6-8 and GRASS-E-6-8 lambs, with the first group having greater FA content (SFA, BCFA, MUFA and PUFA) than the second group. Dim2 separated the animal groups based on the types of fatty acids and the relative abundances of volatile compounds. MIX-W-6-8, WEAN-W-4, REDC-W-6-8 and REDC-E-6-8 showed greater content of PUFA and the associated volatiles than PMER-W-12 and PMER-C-12. CHIC-W-6-8, CHIC-E-6-8, GRASS-W-6-8 and GRASS-E-6-8 were intermediate for their PUFA content and volatiles produced.

## 4. Discussion

The main objective of the present study was to evaluate the meat fatty acid and volatile profiles of lambs from diverse forage-based production systems in New Zealand. Our results indicated that the content of all identified fatty acids and the relative abundance of several volatile compounds (acids: acetic, propanoic, butanoic and hexanoic acids; alcohols: 1-butanol, 1-penten-3-ol, 1-pentanol, 1-hexanol, 1-octen-3-ol, 1-heptanol, 1-octanol and 2-octen-1-ol; aldehydes: 2-methylbutanal, hexanal, octanal and nonanal; ketones: acetone, 2-butanone, 2-pentanone, 2-heptanone, acetoin, butyrolactone; hydrocarbons: Z-2-octene and beta-pinene; furan: 2-ethylfuran; sulphur compounds: dimethyl sulphide and dimethyl sulfone) were affected by the evaluated forage production systems.

### 4.1. Relationships between Production Systems, Fatty Acids and Lipid Volatiles

Lambs slaughtered at weaning clustered with C12:0 and C14:0 in the same quadrant of the PCA plot. The lipid profile of adipose tissue from suckling lambs may reflect the composition of the ingested milk lipids, which contain higher proportions of C12:0 (3.99%), C14:0 (10.17%) and C16:0 (25.10%), and a lower proportion of C18:0 (8.85%) [29], compared to meat lipids. This was in agreement with Bas and Morand-Fehr [30], who observed that fatty acid composition of tissues from milk-fed lambs was characterised by lower percentages of C18:0 and higher percentages of C14:0 and C16:0.

Lambs slaughtered at weaning or that grazed on mixed pasture, red clover or chicory were associated with higher content of PUFA and higher abundance of compounds arising from lipid oxidation, such as 2-heptanone on the top side of the PCA plot. In contrasts, the meat from the 12-month-old Merino lambs had the lowest PUFA and highest SFA, BCFA and total fatty acids content compared with meat from the other animal groups. A negative association between PUFA proportion and total lipid content has been reported in various bovine and ovine studies [31]. The PUFA proportion in meat decreases as animals become older due to the relative increase in tissue deposition of neutral lipids [32]. In the PCA plot, the PUFA were clustered in the same quadrant as 1-penten-3-ol, 1-hexanol, 2-pentanone, 2-methylbutanal, heptanal, 2-heptanone, acetone and 2-butanone (Figure 2), indicating that PUFA are more susceptible to oxidation [15] and contributed to the generation of these aldehydes, ketones and alcohols associated with lipid oxidation volatiles [33].

Carboxylic acids in raw meat can be derived from several pathways, including enzymatic and chemical reactions and from action of spoilage bacteria on lipids, amino acids or carbohydrates [17]. The lower abundance of acetic, propanoic, butanoic and hexanoic acid in meat from 12-month-old Merino lambs is potentially a consequence of differences in their fatty acid profile and associated peroxidation capacity. Acetic acid is present in meat that has been stored under various conditions and it has a high odour threshold of 200 mg/L [34]. Thus, although acetic acid was the acid present in greatest abundance in lamb meat in this study, it was unlikely to have a significant influence on the flavour characteristics. Butanoic acid can be formed from fermentative metabolism during aging and frozen storage in vacuum-packed meat [35], has lower odour threshold (240 μg/L in water) compared to acetic acid and is associated with rancid, sharp and cheesy odours [34]. The lower relative abundance of butanoic acid in meat from 12-month-old Merino lambs suggests lower likelihood of rancid flavour compared to 4- to 8-month-old lambs at weaning or after a period on a forage finishing diet. Pentanoic and heptanoic acids are also associated with imparting fatty, gammy, cheesy and dairy odours and are highly correlated with rancid odour scores according to a trained sensory panel [34,36]. However, these volatiles were present at similar abundance in meat from lambs across the production systems and therefore, were unlikely to contribute to flavour differences among the types of lamb in this study.

The lower abundance of hexanal and octanal in Merino lambs than that in ewe and wether lambs fed on chicory, and wether lambs fed on mixed pasture or red clover, is a potential consequence of their lower PUFA content. Straight-chain aldehydes including hexanal, octanal and nonanal are lipid oxidation products and are characterised to have green-grass and soapy odours [33]. An hexanal to nonanal ratio equal to or lower than 2.45 has been proposed as an indicator of lamb meat freshness and overall quality during storage [33]. Based on this criterion, the lamb meat from all production systems in the current study can be considered as fresh. 1-pentanol is a degradation product of homologous aldehydes such as hexanal during lipid and amino acid oxidation [34]. It is therefore not surprising that meat from Merino lambs also had a lower abundance of 1-pentanol, which has an herbal and fatty odour that has previously been correlated to a rancid odour.

Animal diet is generally considered to be the most important environmental factor affecting the proportion of intramuscular fatty acid composition, especially when comparing meat from pasture- and concentrate-fed animals [35]. Thus, in the present study, the differences in the fatty acid and volatile profiles in meat from diverse forage-production systems were smaller than those reported for meat from pasture- compared to grain-fed animals [37]. Meat from lambs fed chicory showed higher relative abundance of hexanal, octanal and nonanal than meat from lambs fed perennial ryegrass when diets were contrasted within Farm C. The second dimension of the PCA plot also shows that meat from lambs that grazed on chicory had numerically higher abundance of acetoin, which has been associated to oxidation levels in meat from animals fed fat-enriched diets [38] compared to lambs that grazed on perennial ryegrass. The accumulation of acetoin has previously been related to creamy, dairy and cheesy odours, but normally not sufficiently intense to be associated with spoiled or unpleasant odour in meat [39].

No sex or castration effect was found on the fatty acid and volatile profiles when comparisons were made between ewe, wether or cryptorchid lambs fed with the same diet within a farm in the current study. This is not surprising because the ewe and wether lambs had similar carcass characteristics [19] and amount of total fatty acids. The influence of sex on fatty acid composition is mainly due to their differences in the amount of adipose tissue, with females depositing more fat in the carcass than males [13,14], but some studies also suggested that this influence is small in young lambs [40]. The PCA plot also indicated that the effect of sex or castration on fatty acid content and volatile profiles was not significant, as wether and ewe lambs or wether and cryptorchid lambs with the same diet tended to cluster together.

### 4.2. Non-Lipid Volatiles

Some identified volatiles were not generated from lipid oxidation directly and were influenced by the production systems via fatty acid profile differences. Terpenes could be transferred from grass to animal tissue, so these compounds are also considered as green forage indicators [41,42]. For the 6- to 8-month-old lambs, no differences were found in the abundances of beta-pinene and isododecane in meat between lambs grazed on red clover, chicory, perennial ryegrass or mixed pasture. Cornu et al. [43] suggested that beta-pinene in beef could be used to determine the region that an animal came from. Our results indicate that meat hydrocarbons from the forage diets did not differ geographically among the farms investigated in New Zealand.

Sulphur compounds originate from the degradation of cysteine and methionine, two sulphur-containing amino acids [44]. Dimethyl sulfone, which has an unfavourable “milky” flavour in meat from pasture-fed sheep [45], was more abundant in lambs that grazed on red clover compared to lambs that grazed perennial ryegrass, chicory or mixed pasture. This may be a result of different volatiles’ degradation pathways influenced by post-slaughter conditions; under more acidic pH (<6) and oxidised conditions, amino acids and glucose tend to produce more pyrazines and furans instead of sulphur compounds [33]. Dimethyl sulphide, which has a sulphurous, onion, and green odour in raw meat [46], was least abundant in Merino lambs. Unlike straight-chain aldehydes, 2-methylbutanal is a Strecker reaction product from amino acids [47], which had the greatest abundance in MIX-W-6-8 lambs. Frank et al. [48] reported that the abundance of acetone in raw beef increased during aging when the post-mortem proteolysis increased the pool of non-volatile compounds, such as free amino acids and nucleotides. 2-Ethylfuran is formed when amino acids and ribose interact in the lipid oxidation pathway [32] and has a high odour threshold of 8.0 mg/L in water [49]. The ewe lambs that grazed on perennial ryegrass had greater abundance of 2-ethylfuran than wether lambs, but this sex effect was not observed between contrast groups with the other diets.

### 4.3. Nutritional Quality: Selected Fatty Acids, Ratios and Indexes

Excessive intakes of n-6 PUFA together with high n-6:n-3 ratios are commonly found in modern Western diets and associated with pathogenesis of many diseases, including cardiovascular disease, cancer and inflammatory and autoimmune diseases [50]. A dietary n-6:n-3 ratio below 4.0 was reported to reduce total cardiovascular disease mortalities by up to 70% after two years [51]. All lamb production groups used in the current study resulted in a mean n-6:n-3 ratio below 1.5. These ratios are similar to forage-reared lambs from Uruguay reported by Díaz et al. [8] and lower than those found by Gabriella et al. [52] where concentrate feeding was used. The n-6:n-3 ratio, in contrast to the PUFA:SFA ratio, is highly influenced by the fatty acid composition of the diet fed to the animal [30]. Meat from forage-reared lambs has a lower n-6:n-3 ratio than meat from concentrate-reared ones, as C18:3n-3 is the major fatty acid in plant lipids, while C18:2n-6 predominates in concentrate diets [8].

The PUFA:SFA ratios in meat from all production systems in the current study were in the range of 0.17–0.35, which are below the recommended value of ≥0.45 [53]. For human diets, a high SFA intake has been associated with an increase in plasma cholesterol and greater risk of coronary heart disease [54], but not all saturated fatty acids have the same cholesterol-raising potential [55]. Higher amounts of C18:0 in the diet do not elevate plasma low-density lipoprotein-cholesterol because it is poorly digested and can be easily desaturated to C18:1 *cis*-9 [56]. Thus, different fatty acid indexes/ratios have been proposed in addition to the n-6:n-3 and PUFA:SFA ratios to take into account the different impact of fatty acids in human health, such as atherogenic and thrombogenic indexes and h/H and nutritional ratios. Low values of the atherogenic and thrombogenic indexes and the nutritional ratio are recommended for a healthy diet [21], while higher hypocholesterolaemic relative to hypercholesterolaemic fatty acids are beneficial for human health [22]. The atherogenic index range observed in the current study was similar to those found by Salvatori et al. [31], who measured it on lambs reared under extensive conditions and slaughtered at 64 days of age. In the current study, the greater atherogenic index and nutritional ratio and lower h/H ratio of meat from 4-month-old milk-fed lambs was due to the greater proportions of C14:0 and C16:0 fatty acids that are high in milk, as discussed in Section 4.1, whereas the greater thrombogenic index of Merino lambs was due to their lower proportion of PUFA. The thrombogenic index range observed in the current study was similar to previously reported values for lamb, which are slightly greater than pork but lower than milk and cheese [21]. The h/H ratios of 6- to 12-month-old lambs ranged between 1.8 and 2.0, which were similar to the range previously reported for pasture-raised lambs by Santos-Silva et al. [22].

The health benefits obtained by human consumption of meat lipids have been associated with quantitative LC-n-3-PUFA values, mainly EPA and DHA [57]. In the current study, 100 g of fresh muscle of lamb could provide 28 to 44 mg EPA plus DHA, with the greatest concentration coming from the lambs slaughtered at weaning. Similar EPA plus DHA contents in meat from lambs grazed on pasture have been reported by other authors [9,58]. The EPA plus DHA content and proportion in the current study, however, were greater than values reported for lambs fed a concentrate diet [59], or even when concentrate-fed lambs were supplemented with linseed [60].

Meat from almost all lambs used in the current study could be considered as a “source” of n-3 fatty acids (EPA plus DHA) by the Australia and New Zealand reference standard [10], but only the meat from lambs which were slaughtered at weaning met the Australia and New Zealand standard to be considered as a “good source” of n-3. However, the potential to aim for nutritional claims on EPA plus DHA is limited by total SFA plus *trans*-fatty acids in Australia and New Zealand and would depend on the nutritional guidelines and regulations of the export market destinations. When the European standards are considered [11], only meat from lambs slaughtered at weaning or finished on mixed pasture could be considered as a “source” of n-3, with none of the production systems providing meat that obtained the reference standard of a “good source” of n-3 fatty acids.

The proportions of the CLA isomer C18:2 *cis*-9, *trans*-11 in lamb loin samples in the current study were similar to previously reported values in loin muscle tissue of extensively finished commercial lambs [9]. The CLA isomer *cis*-9, *trans*-11 found in meat has been associated with anticarcinogenic, antiatherogenic and antidiabetic properties [61], but nutrition guidelines do not offer a dietary reference value [11] as the potential health benefits in humans associated with CLA intake are still a topic of debate.

## 5. Conclusions

Meat from 12-month-old Merino lambs had low abundance of straight-chain volatile acids and aldehydes, including acetic acid, butanoic acid, hexanoic acid, hexanal and octanal, as a consequence of its lower PUFA content. Carbon disulphide that originated from amino acids degradation was in similar abundance in meat from all production systems. Lambs slaughtered at weaning or further grazed on red clover, chicory or mixed pasture may result in distinctive meat flavour due to greater abundance of overall meat volatiles compared with meat from lambs that grazed on perennial ryegrass or meat from 12-month-old Merino lambs.

Finishing lambs for a longer period of up to 12 months in New Zealand forage systems will result in a lower proportion of n-3 fatty acids in meat as well as a lower PUFA:SFA ratio compared to 4- to 8-month-old lambs. The combined EPA plus DHA content in muscle and the proportion of lambs that can be considered a ‘source’ or ‘good source’ of these target fatty acids differed significantly among the ten evaluated forage-based production systems. However, the potential to aim for nutritional claims on EPA plus DHA is limited by total SFA plus *trans* fatty acids and would depend on the nutritional guidelines and regulations of the export markets.

## Figures and Tables

**Table 1:** Animal genetics, sex, diet and age at slaughter and distance to meat plant for Farms A, B and C (*n* = 15 lambs per group).

Production System	Farm	Approximate Age at Slaughter (Months)	Sex	Diet ^1^	Genetics
REDC-W-6-8	A	6–8	Wethers	Red Clover	Perendale × LambSupreme ^2^
REDC-E-6-8	A	6–8	Ewes	Red Clover	Perendale × LambSupreme ^2^
MIX-W-6-8	A	6–8	Wethers	Mixed pasture	Perendale × Romney
PMER-W-12	B	12	Wethers	Pasture	Merino
PMER-C-12	B	12	Cryptorchids	Pasture	Merino
GRASS-W-6-8	C	6–8	Wethers	Pasture	Composite ^3^
GRASS-E-6-8	C	6–8	Ewes	Pasture	Composite ^3^
CHIC-W-6-8	C	6–8	Wethers	Chicory	Composite ^3^
CHIC-E-6-8	C	6–8	Ewes	Chicory	Composite ^3^
WEAN-W-4	C	4	Wethers	Pre-weaning	Composite ^3^

^1^ Farm A: red clover (*Trifolium pratense*); ^1^ Farm A-Mixed pasture: perennial ryegrass-red clover/white clover mix; ^1^ Farm B: perennial ryegrass (*Lolium perenne*) and white clover (*Trifolium repens*) mix, followed by fescue (*Lolium arundinaceum*), red and white clover and plantain (*Plantago lanceolata*) mix during the last 2 weeks; ^1^ Farm C: predominantly Italian (*Lolium multiflorum*) and perennial ryegrass, and red and white clover mix; ^1^ Farm C-Pre-weaning: suckled and grazing mothers’ diet of a chicory (*Cichorium intybus*) and red clover mix; ^2^ LambSupreme: lean-selected Poll Dorset, Wiltshire, Romney x Dorset, Coopworth, Texel, and high-growth Romney; ^3^ Composite: Perendale, Texel, Finnish Landrace and Romney.

**Table 2:** Fatty acid content (mg/100 g raw meat) of *Longissimus thoracis* from lambs reared under different New Zealand commercial forage production systems.

Fatty Acids	4 Months Old	6–8 Months Old							12 Months Old		SEM ^10^	*p*-Value
	WEAN-W-4	GRASS-W-6-8	GRASS-E-6-8	CHIC-W-6-8	CHIC-E-6-8	REDC-W-6-8	REDC-E-6-8	MIX-W-6-8	PMER-W-12	PMER-C-12		
C10:0	6.64 ^c^	3.09 ^ab^	3.57 ^ab^	2.97 ^ab^	2.73 ^a^	3.17 ^ab^	3.09 ^ab^	3.73 ^ab^	4.31 ^b^	3.94 ^ab^	0.448	<0.001
C12:0	12.31 ^c^	6.23 ^ab^	6.05 ^ab^	4.96 ^ab^	7.00 ^b^	5.89 ^ab^	3.37 ^a^	5.45 ^ab^	4.03 ^ab^	3.47 ^ab^	1.097	<0.001
C14:0	122.15 ^b^	47.69 ^a^	52.23 ^a^	45.51 ^a^	41.49 ^a^	56.82 ^a^	46.82 ^a^	66.08 ^a^	69.03 ^a^	56.69 ^a^	9.76	<0.001
C15:0	13.08 ^d^	6.95 ^ab^	7.11 ^ab^	6.08 ^a^	5.31 ^a^	7.94 ^ab^	6.53 ^ab^	8.67 ^abc^	12.03 ^cd^	9.95 ^bcd^	1.107	<0.001
C16:0	645.84 ^bc^	423.61 ^a^	499.05 ^ab^	458.19 ^ab^	431.67 ^a^	503.66 ^ab^	497.20 ^ab^	579.09 ^abc^	733.65 ^c^	634.20 ^bc^	62.20	<0.001
C17:0	26.55 ^bc^	20.55 ^ab^	21.28 ^ab^	19.17 ^ab^	17.50 ^a^	22.99 ^abc^	21.63 ^ab^	25.95 ^abc^	37.37 ^d^	30.65 ^cd^	2.660	<0.001
C18:0	349.26 ^ab^	320.44 ^a^	333.71 ^a^	308.54 ^a^	297.93 ^a^	362.39 ^ab^	351.25 ^ab^	414.19 ^ab^	557.91 ^c^	468.19 ^bc^	40.48	<0.001
C20:0 ^1^	0.66 ^a^	0.01 ^a^	0.15 ^a^	bd	bd	0.59 ^a^	0.22 ^a^	0.53 ^a^	3.48 ^b^	2.69 ^b^	0.285	<0.001
C22:0	bd	2.19 ^c^	0.87 ^a^	1.18 ^ab^	2.11 ^c^	bd	bd	bd	1.22 ^ab^	1.79 ^bc^	0.223	<0.001
C24:0	1.88 ^b^	bd	bd	bd	bd	1.39 ^a^	1.43 ^a^	1.35 ^a^	bd	bd	0.095	<0.001
iso-C14:0	bd	0.20 ^a^	0.09 ^ab^	bd	bd	bd	bd	bd	0.63 ^b^	0.40 ^ab^	0.145	<0.001
iso-C15:0	2.67 ^cd^	2.37 ^ab^	2.07 ^ab^	1.65 ^ab^	1.05 ^a^	2.17 ^abc^	1.56	2.46	4.38	3.71	0.464	<0.001
anteiso-C15:0	5.09 ^cd^	3.23 ^ab^	3.15 ^ab^	2.75 ^ab^	2.10 ^a^	3.50 ^abc^	2.70 ^a^	3.60 ^abc^	5.56 ^d^	4.30 ^bcd^	0.499	<0.001
iso-C16:0	3.91	1.98	1.86	1.78	1.75	2.69	2.23	2.12	2.16	2.16	0.310	<0.001
iso-C17:0	9.47 ^cd^	7.57 ^abc^	7.27 ^abc^	6.00 ^ab^	5.01 ^a^	8.31 ^bc^	7.23 ^abc^	9.64 ^cd^	13.19 ^e^	11.47 ^de^	0.938	<0.001
anteiso-C17:0	13.01 ^cd^	8.45 ^ab^	8.86 ^abc^	7.90 ^ab^	9.09 ^a^	8.11 ^abc^	10.85 ^ab^	10.85 ^bcd^	17.73 ^e^	13.75 ^de^	1.326	<0.001
BCFA ^2^	34.14 ^cde^	23.82 ^abc^	23.33 ^abc^	20.09 ^ab^	16.24 ^a^	25.78 ^abcd^	21.82 ^ab^	29.42 ^bcd^	43.59 ^e^	35.77 ^de^	3.416	<0.001
SFA ^3^	1213.86 ^bcd^	855.57 ^ab^	947.21 ^abc^	866.63^a b^	821.99 ^a^	990.07 ^abc^	953.16 ^abc^	1133.91 ^abcd^	1463.15 ^d^	1244.67 ^cd^	116.1	<0.001
C14:1	4.07 ^b^	1.00 ^a^	1.14 ^a^	0.81 ^a^	0.75 ^a^	1.51 ^a^	1.17 ^a^	1.91 ^a^	1.46 ^a^	1.22 ^a^	0.430	<0.001
C16:1	35.82 ^c^	19.53 ^a^	23.70 ^ab^	19.94 ^a^	18.85 ^a^	23.85 ^ab^	22.85 ^ab^	27.99 ^abc^	34.85 ^c^	31.84 ^bc^	3.271	<0.001
C17:1	13.57 ^b^	3.73 ^a^	2.42 ^a^	3.90 ^a^	2.85 ^a^	10.93 ^b^	11.95 ^b^	14.15 ^b^	2.45 ^a^	3.47 ^a^	1.112	<0.001
C18:1 *trans*-9	5.41 ^c^	3.77 ^ab^	3.73 ^ab^	3.87 ^ab^	3.33 ^a^	4.77 ^ab^	4.50 ^ab^	5.29 ^bc^	6.73 ^c^	5.52 ^bc^	0.606	<0.001
C18:1 *trans*-11	73.36 ^c^	42.34 ^a^	48.71 ^ab^	54.54 ^abc^	45.91 ^ab^	69.80 ^abc^	62.51 ^abc^	66.83 ^abc^	78.06 ^c^	54.09 ^abc^	9.09	<0.001
C18:1 *cis*-9	816.06 ^abc^	672.16 ^ab^	742.11 ^abc^	676.69 ^ab^	625.41 ^a^	755.61 ^abc^	783.97 ^abc^	964.95 ^bcd^	1249.19 ^d^	1041.75 ^cd^	95.3	<0.001
C18:1 *cis*-11	27.56 ^bc^	20.26 ^a^	21.47 ^a^	21.09 ^a^	19.29 ^a^	24.58 ^ab^	24.28 ^ab^	27.98 ^bc^	31.39 ^c^	28.35 ^bc^	1.891	<0.001
C24:1	bd	0.01 ^c^	0.01 ^ab^	0.01 ^bc^	0.01 ^bc^	bd	bd	bd	0.01 ^c^	0.01 ^bc^	0.179	<0.001
MUFA^4^	975.88 ^abc^	762.79 ^ab^	843.23^abc^	780.85 ^ab^	716.39 ^a^	891.04 ^abc^	911.27 ^abc^	1109.14 ^bcd^	1404.15 ^d^	1166.15 ^cd^	108.0	<0.001
C18:2n-6	148.49 ^e^	92.03 ^abc^	92.63 ^abc^	119.99 ^e^	115.69 ^de^	105.13 ^cde^	103.34 ^cde^	98.32 ^bcd^	80.28 ^ab^	74.76 ^a^	6.15	<0.001
C18:2 *cis*-9, *trans*-11	35.78 ^b^	18.73 ^a^	20.65 ^a^	22.45 ^ab^	18.64 ^a^	26.67 ^b^	23.93 ^ab^	30.44 ^ab^	31.43 ^ab^	21.35 ^a^	4.179	<0.001
C20:4n-6	36.85 ^ab^	37.05 ^ab^	31.46 ^a^	32.29 ^ab^	30.12 ^ab^	38.00 ^b^	36.29 ^ab^	38.21 ^b^	33.65 ^ab^	31.55 ^ab^	2.311	<0.001
C22:2n-6	0.78 ^b^	bd	bd	bd	bd	0.45 ^ab^	0.13 ^a^	bd	bd	0.17 ^a^	0.165	<0.001
C18:3n-3	84.05 ^d^	46.43 ^ab^	49.35 ^abc^	61.21 ^c^	58.16 ^bc^	61.44 ^c^	59.16 ^bc^	58.10 ^bc^	46.01 ^ab^	42.48 ^a^	4.167	<0.001
C20:5n-3	33.03 ^d^	24.65 ^ab^	23.92 ^ab^	26.29 ^bc^	25.05 ^ab^	30.87 ^cd^	30.44 ^cd^	31.89 ^d^	21.32 ^a^	21.54 ^ab^	1.491	<0.001
C22:5n-3	31.59 ^c^	23.94 ^a^	22.41 ^a^	23.37 ^a^	22.27 ^a^	28.29 ^bc^	27.51 ^b^	29.35 ^bc^	22.20 ^a^	21.83 ^a^	1.055	<0.001
C22:6n-3	10.49 ^d^	7.49 ^abc^	7.21 ^abc^	6.87 ^ab^	6.31 ^a^	8.47 ^bc^	8.15 ^abc^	8.93 ^cd^	6.43 ^a^	6.47 ^a^	0.587	<0.001
PUFA ^5^	381.08 ^e^	250.32 ^abc^	247.64 ^abc^	292.57 ^cd^	276.23 ^bcd^	299.33 ^d^	288.97 ^bcd^	295.25 ^cd^	241.32 ^ab^	220.17 ^a^	15.04	<0.001
EPA+DHA ^6^	43.52 ^c^	32.13 ^a^	31.13 ^a^	33.15 ^ab^	31.35 ^a^	39.34 ^bc^	38.59 ^bc^	40.82 ^c^	27.77 ^a^	28.01 ^a^	1.934	<0.001
n-6 PUFA sum ^7^	185.35 ^e^	129.09 ^abcd^	124.07 ^abc^	152.38 ^d^	145.81 ^cd^	143.13 ^cd^	139.64 ^cd^	136.54 ^bcd^	113.93 ^ab^	106.33 ^a^	7.61	<0.001
n-3 PUFA sum ^8^	159.18 ^d^	101.54 ^ab^	103.73 ^ab^	117.74 ^bc^	111.77 ^abc^	129.08 ^c^	125.27 ^c^	128.28 ^c^	95.97 ^a^	92.31 ^a^	6.26	<0.001
LC n-3 PUFA ^9^	75.11 ^c^	56.60 ^a^	55.02 ^a^	57.76 ^a^	54.66 ^a^	67.75 ^bc^	67.75 ^b^	70.17 ^bc^	51.31 ^a^	50.75 ^a^	2.805	<0.001
Unreported	223.00 ^bc^	187.44 ^ab^	190.99 ^ab^	177.62 ^a^	174.03 ^a^	214.70 ^abc^	197.75 ^ab^	222.63 ^bc^	243.98 ^c^	223.42 ^bc^	12.95	<0.001
Total FA	2792.48 ^bcd^	2084.40 ^ab^	2241.72 ^abc^	2118.91 ^abc^	1989.71 ^a^	2395.82 ^abc^	2351.36 ^abc^	2761.45 ^abcd^	3357.82 ^d^	2857.42 ^cd^	243.5	<0.001

^a, b, c, d, e^ Different letters within the same row denote significant difference between means (*p* < 0.05). ^1^ bd: below detection threshold of 0.01 mg/100 g raw meat. ^2^ BCFA = ∑ iso-C14:0, iso-C15:0, anteiso-C15:0, iso-C16:0, iso-C17:0, anteiso-C17:0. ^3^ SFA = ∑ C10:0, C12:0, C14:0, C15:0, C16:0, C17:0, C18:0, C20:0, C22:0, C24:0, iso-C14:0, iso-C15:0, anteiso-C15:0, iso-C16:0, iso-C17:0, anteiso-C17:0. ^4^ MUFA = ∑ C14:1, C16:1, C17:1, C18:1 *trans*-9, C18:1 *trans*-11, C18:1 *cis*-9, C18:1 *cis*-11, C20:1, C22:1, C24:1. ^5^ PUFA = ∑ C18:2n-6, C20:4n-6, C22:2 n6, C18:3n-3, C20:5 n3, C22:5 n3, C22:6n-3, C18:2 *cis*-9, *trans*-11. ^6^ EPA + DHA = ∑ C20:5n-3, C22:6n-3. ^7^ n-6 PUFA = ∑ C18:2n-6, C20:4n-6, C22:2n-6. ^8^ n-3 PUFA = ∑ C18:3n-3, C20:5n-3, C22:5n-3, C22:6n-3. ^9^ LC n-3 PUFA = ∑ C20:5n-3, C22:5n-3, C22:6n-3. ^10^ SEM: standard error of the means.

**Table 3:** Fatty acid composition of *Longissimus thoracis* as a percentage of total fatty acids from lambs reared under different New Zealand commercial production systems.

Fatty Acids	4 Months Old	6–8 Months Old							12 Months Old		SEM ^9^	*p*-Value
	WEAN-W-4	GRASS-W-6-8	GRASS-E-6-8	CHIC-W-6-8	CHIC-E-6-8	REDC-W-6-8	REDC-E-6-8	MIX-W-6-8	PMER-W-12	PMER-C-12		
C10:0	0.23 ^c^	0.15 ^ab^	0.16 ^b^	0.14 ^ab^	0.14 ^ab^	0.13 ^ab^	0.13 ^a^	0.13 ^ab^	0.13 ^a^	0.14 ^ab^	0.009	<0.001
C12:0	0.43 ^e^	0.33 ^cde^	0.27 ^bcde^	0.24 ^abcd^	0.38 ^de^	0.24 ^abcd^	0.14 ^ab^	0.19 ^abc^	0.12 ^a^	0.12 ^a^	0.047	<0.001
C14:0	4.26 ^b^	2.31 ^a^	2.32 ^a^	2.14 ^a^	2.02 ^a^	2.24 ^a^	1.95 ^a^	2.34 ^a^	2.06 ^a^	1.91 ^a^	0.215	<0.001
C15:0	0.46 ^e^	0.34 ^cd^	0.32 ^abcd^	0.29 ^abc^	0.27^a^	0.32 ^abcd^	0.28 ^ab^	0.31 ^abcd^	0.36 ^d^	0.34 ^bcd^	0.021	<0.001
C16:0	23.01 ^b^	20.42 ^a^	22.05 ^ab^	21.39 ^ab^	21.43 ^ab^	20.59 ^a^	20.89 ^a^	20.82 ^a^	21.74 ^ab^	21.91 ^ab^	0.548	<0.001
C17:0	0.95 ^ab^	0.99 ^bc^	0.96 ^ab^	0.91 ^a^	0.88 ^a^	0.95 ^ab^	0.92 ^ab^	0.94 ^ab^	1.11 ^d^	1.07 ^cd^	0.027	<0.001
C18:0	12.61 ^a^	15.55 ^bcd^	15.00 ^bc^	14.56 ^b^	14.99 ^bc^	14.99 ^bc^	14.89 ^b^	15.01 ^bc^	16.61 ^d^	16.38 ^cd^	0.441	<0.001
C20:0	0.0229	0.0003	0.0051	-	-	0.0214	0.0080	0.0181	0.1118	0.0971	0.008	<0.001
C22:0	-	0.1119 ^cd^	0.0624 ^b^	0.0634 ^b^	0.1237 ^d^	-	-	-	0.0385 ^ab^	0.0753 ^bc^	0.012	<0.001
C24:0	0.0757 ^c^	-	-	-	-	0.0717 ^bc^	0.0710 ^bc^	0.0555 ^b^	-	-	0.006	<0.001
iso-C14:0	-	0.0119 ^ab^	0.0039 ^a^	-	-	-	-	-	0.0180 ^b^	0.0116 ^ab^	0.005	<0.001
iso-C15:0	0.10 ^bcd^	0.13 ^cd^	0.11 ^bcd^	0.09 ^abc^	0.05 ^a^	0.09 ^abc^	0.07 ^ab^	0.09 ^abcd^	0.14 ^d^	0.14 ^d^	0.014	<0.001
anteiso-C15:0	0.18 ^d^	0.16 ^cd^	0.15 ^bcd^	0.13 ^abc^	0.10 ^a^	0.14 ^abcd^	0.11 ^ab^	0.13 ^abc^	0.17 ^cd^	0.15 ^bcd^	0.013	<0.001
iso-C16:0	0.14 ^c^	0.10 ^b^	0.09 ^ab^	0.09 ^ab^	0.09 ^ab^	0.11 ^b^	0.09 ^ab^	0.10 ^b^	0.07 ^a^	0.08 ^ab^	0.009	<0.001
iso-C17:0	0.33 ^bcd^	0.37 ^cde^	0.33 ^bc^	0.29 ^ab^	0.25 ^a^	0.35 ^bcde^	0.31 ^ab^	0.35 ^bcde^	0.39 ^de^	0.40 ^e^	0.019	<0.001
anteiso-C17:0	0.46 ^def^	0.41 ^cde^	0.40 ^bcd^	0.37 ^abc^	0.31^a^	0.37 ^abc^	0.34 ^ab^	0.39 ^bc^	0.53 ^f^	0.48 ^ef^	0.021	<0.001
BCFA ^1^	1.20 ^cd^	1.17 ^cd^	1.07 ^bc^	0.95 ^ab^	0.81 ^a^	1.04 ^bc^	0.92 ^ab^	1.06 ^bc^	1.30 ^d^	1.25 ^cd^	0.067	<0.001
SFA ^2^	43.24 ^bc^	41.38 ^abc^	42.20 ^abc^	40.68 ^ab^	41.01 ^ab^	40.58 ^ab^	40.19 ^a^	40.86 ^ab^	43.56 ^c^	43.28 ^c^	0.697	<0.001
C14:1	0.14 ^b^	0.05 ^a^	0.05 ^a^	0.04 ^a^	0.03 ^a^	0.06 ^a^	0.05 ^a^	0.07 ^a^	0.04 ^a^	0.04 ^a^	0.014	<0.001
C16:1	1.26 ^c^	0.94 ^ab^	1.04 ^ab^	0.92 ^a^	0.94 ^a^	0.97 ^ab^	0.96 ^ab^	1.00 ^ab^	1.04 ^ab^	1.12 ^bc^	0.055	<0.001
C17:1	0.48 ^c^	0.19 ^b^	0.11 ^ab^	0.19 ^b^	0.15 ^ab^	0.47 ^c^	0.51 ^c^	0.52 ^c^	0.08 ^a^	0.12 ^ab^	0.031	<0.001
C18:1 *trans*-9	0.19 ^ab^	0.18 ^ab^	0.16 ^a^	0.18 ^ab^	0.16 ^a^	0.19 ^ab^	0.19 ^ab^	0.19 ^ab^	0.20 ^b^	0.19 ^ab^	0.010	<0.001
C18:1 *trans*-11	2.62 ^b^	2.08 ^ab^	2.19 ^ab^	2.53 ^ab^	2.24 ^ab^	2.78 ^b^	2.65 ^b^	2.40 ^ab^	2.33 ^ab^	1.89 ^a^	0.214	<0.001
C18:1 *cis*-9	29.21 ^a^	32.26 ^abc^	32.70 ^bc^	31.55 ^ab^	31.06 ^ab^	31.15 ^ab^	33.02 ^bc^	34.74 ^cd^	37.04 ^d^	36.17 ^d^	0.953	<0.001
C18:1 *cis*-11	1.00 ^ab^	1.00 ^ab^	0.98 ^ab^	1.01 ^ab^	0.99 ^ab^	1.06 ^ab^	1.04 ^ab^	1.02 ^ab^	0.94 ^a^	1.01 ^ab^	0.033	<0.001
C24:1	-	0.0801 ^e^	0.0339 ^bc^	0.0655 ^de^	0.0575 ^cde^	-	-	-	0.0440 ^cd^	0.0383 ^cd^	0.009	<0.001
MUFA ^3^	34.89 ^a^	36.76 ^abc^	37.26 ^bcd^	36.48 ^abc^	35.62 ^ab^	36.67 ^abc^	38.42 ^cde^	39.94 ^def^	41.72 ^f^	40.57 ^ef^	0.935	<0.001
C18:2n-6	5.41 ^def^	4.66 ^cd^	4.42 ^cd^	5.85 ^ef^	6.03 ^f^	4.72 ^cde^	4.57 ^cd^	3.63 ^bc^	2.46 ^a^	2.71 ^ab^	0.353	<0.001
C18:2 *cis*-9, *trans*-11	1.25 ^c^	0.91 ^ab^	0.92 ^ab^	1.03 ^abc^	0.90 ^ab^	1.05 ^abc^	1.00 ^abc^	1.08 ^bc^	0.94 ^abc^	0.74 ^a^	0.100	<0.001
C20:4n-6	1.36 ^abc^	1.90 ^d^	1.52 ^abcd^	1.60 ^bcd^	1.58 ^abcd^	1.77 ^cd^	1.62 ^bcd^	1.45 ^abcd^	1.04 ^a^	1.18 ^ab^	0.172	<0.001
C22:2n-6	0.0289 ^b^	-	-	-	-	0.0142 ^ab^	0.0053 ^ab^	-	-	0.0068 ^ab^	0.005	<0.001
C18:3n-3	3.03 ^d^	2.30 ^bc^	2.32 ^bc^	2.99 ^d^	3.03 ^d^	2.71 ^cd^	2.59 ^bcd^	2.13 ^b^	1.39 ^a^	1.52 ^a^	0.462	<0.001
C20:5n-3	1.21 ^d^	1.23 ^bc^	1.13 ^bc^	1.30 ^d^	1.33 ^d^	1.43 ^cd^	1.37 ^bcd^	1.21 ^b^	0.66 ^a^	0.82 ^a^	0.169	<0.001
C22:5n-3	1.16 ^c^	1.21 ^bc^	1.06 ^bc^	1.14 ^c^	1.18 ^c^	1.30 ^c^	1.23 ^c^	1.11 ^c^	0.68 ^a^	0.82 ^ab^	0.119	<0.001
C22:6n-3	0.38 ^c^	0.38 ^c^	0.34 ^bc^	0.34 ^bc^	0.33 ^bc^	0.39 ^c^	0.37 ^c^	0.34 ^bc^	0.20 ^a^	0.24 ^ab^	0.036	<0.001
PUFA ^4^	13.83 ^c^	12.59 ^bc^	11.70 ^bc^	14.24 ^c^	14.37 ^c^	13.38 ^bc^	12.76 ^bc^	10.95 ^b^	7.36 ^a^	8.04 ^a^	0.821	<0.001
EPA+DHA ^5^	1.59 ^c^	1.61 ^c^	1.47 ^bc^	1.64 ^c^	1.66 ^c^	1.82 ^c^	1.74 ^c^	1.55 ^c^	0.86 ^a^	1.06 ^ab^	0.151	<0.001
n-6 PUFA ^6^	6.80 ^cd^	6.56 ^bcd^	5.94 ^bc^	7.44 ^cd^	7.62 ^d^	6.50 ^bcd^	6.20 ^bcd^	5.08 ^ab^	3.50 ^a^	3.90 ^a^	0.501	<0.001
n-3 PUFA ^7^	5.78 ^b^	5.12 ^b^	4.85 ^b^	5.77 ^b^	5.86 ^b^	5.83 ^b^	5.56 ^b^	4.79 ^b^	2.93 ^a^	3.41 ^a^	0.390	<0.001
LC n-3PUFA ^8^	2.75 ^c^	2.82 ^c^	2.53 ^bc^	2.78 ^c^	2.83 ^c^	3.12 ^c^	2.97 ^c^	2.66 ^bc^	1.54 ^a^	1.88 ^ab^	0.146	<0.001
Unreported	8.04 ^ab^	9.28 ^a^	8.84 ^a^	8.60 ^ab^	9.00 ^a^	9.67 ^a^	8.63 ^ab^	8.25 ^ab^	7.36 ^a^	8.12 ^ab^	0.425	<0.001

^a, b, c, d, e, f^ Different letters within the same row denote significant difference between means (*p* < 0.05). ^1^ BCFA = ∑ iso-C14:0, iso-C15:0, anteiso-C15:0, iso-C16:0, iso-C17:0, anteiso-C17:0. ^2^ SFA = ∑ C10:0, C12:0, C14:0, C15:0, C16:0, C17:0, C18:0, C20:0, C22:0, C24:0, iso-C14:0, iso-C15:0, anteiso-C15:0, iso-C16:0, iso-C17:0, anteiso-C17:0. ^3^ MUFA = ∑ C14:1, C16:1, C17:1, C18:1 *trans*-9, C18:1 *trans*-11, C18:1 *cis*-9, C18:1 *cis*-11, C20:1, C22:1, C24:1. ^4^ PUFA = ∑ C18:2n-6, C20:4n-6, C22:2 n6, C18:3n-3, C20:5 n3, C22:5 n3, C22:6n-3, C18:2 *cis*-9, *trans*-11. ^5^ EPA + DHA = ∑ C20:5n-3, C22:6n-3. ^6^ n-6 PUFA = ∑ C18:2n-6, C20:4n-6, C22:2n-6. ^7^ n-3 PUFA = ∑ C18:3n-3, C20:5n-3, C22:5n-3, C22:6n-3. ^8^ LC n-3 PUFA = ∑ C20:5n-3, C22:5n-3, C22:6n-3. ^9^ SEM: standard error of the means.

**Table 4:** Ratio of fatty acids and indexes related to human health in intramuscular fat of *Longissimus thoracis* from lambs reared under different commercial forage production systems.

Atty Acids	4 Months Old	6–8 Months Old							12 Months Old		SEM ^5^	*p*-Value
	WEAN-W-4	GRASS-W-6-8	GRASS-E-6-8	CHIC-W-6-8	CHIC-E-6-8	REDC-W-6-8	REDC-E-6-8	MIX-W-6-8	PMER -W-12	PMER -C-12		
PUFA:SFA	0.32 ^bc^	0.31 ^bc^	0.28 ^bc^	0.35 ^c^	0.35 ^c^	0.33 ^bc^	0.32 ^bc^	0.27 ^b^	0.17 ^a^	0.19 ^a^	0.02	<0.001
n-6:n-3	1.17 ^abc^	1.30 ^bc^	1.23 ^abc^	1.30 ^bc^	1.32 ^c^	1.11 ^ab^	1.12 ^abc^	1.06 ^a^	1.19 ^abc^	1.15 ^abc^	0.06	<0.001
Thrombogenic index ^1^	0.97 ^a^	0.95 ^a^	0.98 ^a^	0.89 ^a^	0.90 ^a^	0.89 ^a^	0.89 ^a^	0.95 ^a^	1.19 ^b^	1.14 ^b^	0.05	<0.001
Atherogenic index ^2^	0.86 ^b^	0.62 ^a^	0.66 ^a^	0.61 ^a^	0.61 ^a^	0.61 ^a^	0.58 ^a^	0.61 ^a^	0.63 ^a^	0.62 ^a^	0.03	<0.001
Nutritional ratio ^3^	0.80 ^b^	0.63 ^a^	0.66 ^a^	0.64 ^a^	0.64 ^a^	0.64 ^a^	0.61 ^a^	0.61 ^a^	0.61 ^a^	0.62 ^a^	0.01	<0.001
hypocholesterolaemic/Hypercholesterolaemic ratio ^4^	1.54 ^a^	1.94 ^b^	1.80 ^b^	1.91 ^b^	1.92 ^b^	1.92 ^b^	1.98 ^b^	1.93 ^b^	1.83 ^b^	1.84 ^b^	0.02	<0.001

^a, b, c^ Different letters within the same row denote significant difference between means (*p* < 0.05). ^1^ Thrombogenic index = (C14:0 + C16:0 + C18:0)/((0.5 × ∑MUFA) + (0.5 × ∑n-6 PUFA) + (3 × ∑n-3 PUFA) + (n-3/n-6)) [21]. ^2^ Atherogenic index = (C12:0 + (4 × C14:0) + C16:0)/((∑n-6 PUFA) + (∑n-3 PUFA) + (∑MUFA)) [21]. ^3^ Nutritional ratio = (C12:0 + C14:0 + C16:0)/(C18:1 + C18:2) [23]. ^4^ hypocholesterolaemic/Hypercholesterolaemic ratio = (C18:1 cis-9 + C18:2n-6 + C20:4n-6 + C18:3n-3 + C20:5n-3 + C22:5n-3 + C22:6n-3)/(C14:0 + C16:0) [22]. ^5^ SEM: standard error of the means.

**Table 5:** Volatile compounds in the headspace of raw lamb loin from animals finished on different forage systems. The values for different volatile compounds correspond to the peak area of the selected mass ion (ion used) divided by the peak area of the internal standard ion (*m/z 81*).

Compounds	Calculated RI ^1^	Ion Used	4 Months Old	6–8 Months Old								12 Months Old	SEM ^2^	*p*-Value
			WEAN-W-4	GRASS-W-6-8	GRASS-E-6-8	CHIC-W-6-8	CHIC-E-6-8	REDC-W-6-8	REDC-E-6-8	MIX-W-6-8	PMER-W-12	PMER-C-12		
**Acids**														
Acetic acid	1483	43	6.29 ^b^	5.55 ^ab^	5.93 ^b^	6.19 ^b^	6.62 ^b^	6.06 ^b^	6.27 ^b^	5.48 ^ab^	4.46 ^ab^	3.60 ^a^	0.48	0.010
Propanoic acid	1566	74	0.41 ^b^	0.31 ^ab^	0.35 ^ab^	0.41 ^b^	0.39 ^b^	0.40 ^b^	0.35 ^ab^	0.45 ^b^	0.23 ^a^	0.20 ^a^	0.03	<0.001
Butanoic acid	1654	60	3.43 ^c^	3.41 ^c^	2.28 ^abc^	2.82 ^bc^	3.02 ^c^	3.32 ^c^	3.16 ^c^	2.98 ^c^	1.54 ^ab^	1.33 ^a^	0.30	<0.001
Pentanoic acid	1765	86	0.91	0.18	0.22	0.09	0.32	0.12	0.24	0.16	0.20	0.09	0.19	0.740
Hexanoic acid	1872	60	3.43 ^bcd^	2.72 ^abc^	2.77 ^abc^	4.03 ^d^	3.59 ^cd^	3.35 ^abcd^	3.12 ^abcd^	4.13 ^d^	2.43 ^ab^	2.28 ^a^	0.24	<0.001
Heptanoic acid	1979	60	0.43	0.32	0.40	0.41	0.39	0.38	0.41	0.44	0.33	0.33	0.03	0.336
Octanoic acid	2086	60	0.80	0.47	0.64	0.64	0.55	0.55	0.55	0.61	0.56	0.58	0.05	0.059
Nonanoic acid	2193	73	0.34	0.25	0.35	0.37	0.40	0.34	0.50	0.44	0.30	0.33	0.05	0.180
**Alcohols**														
1-Butanol	1140	56	0.66 ^abcd^	0.36 ^a^	0.39 ^a^	0.52 ^abc^	0.47 ^ab^	0.52 ^abc^	0.42 ^a^	0.97 ^d^	0.85 ^bcd^	0.93 ^cd^	0.09	<0.001
1-Penten-3-ol	1155	57	6.47 ^ab^	5.92 ^ab^	5.37 ^a^	11.56 ^cd^	9.87 ^bc^	11.56 ^cd^	8.70 ^abc^	14.49 ^d^	4.70 ^a^	6.20 ^ab^	0.89	<0.001
1-Pentanol	1244	42	2.69 ^a^	4.06 ^ab^	3.47 ^a^	6.61 ^c^	5.91 ^bc^	5.54 ^bc^	4.46 ^ab^	7.12 ^c^	2.70 ^a^	3.38 ^a^	0.43	<0.001
1-Hexanol	1347	56	1.42 ^a^	1.41 ^a^	1.41 ^a^	2.66 ^cd^	2.36 ^abcd^	2.63 ^bcd^	1.97 ^abc^	3.31 ^d^	1.54 ^ab^	2.02 ^abc^	0.25	<0.001
2,5-Hexanediol	1418	43	0.13	0.13	0.14	0.15	0.16	0.14	0.14	0.14	0.13	0.14	0.01	0.945
1-Octen-3-ol	1442	57	4.07 ^ab^	4.54 ^ab^	3.57 ^a^	8.01 ^c^	6.64 ^bc^	6.39 ^bc^	4.72 ^ab^	8.27 ^c^	3.64 ^a^	4.50 ^ab^	0.60	<0.001
1-Heptanol	1449	70	0.27 ^a^	0.46 ^abc^	0.48 ^abc^	0.68 ^cd^	0.67 ^cd^	0.75 ^de^	0.59 ^bcd^	1.00 ^e^	0.38 ^ab^	0.50 ^abcd^	0.06	<0.001
1-Octanol	1552	56	0.26 ^a^	0.42 ^abc^	0.46 ^abcd^	0.54 ^bcd^	0.58 ^cd^	0.65 ^de^	0.52 ^bcd^	0.80 ^e^	0.34 ^ab^	0.42 ^abc^	0.04	<0.001
2-Octen-1-ol	1608	57	0.17 ^ab^	0.20 ^ab^	0.15 ^a^	0.31 ^c^	0.25 ^bc^	0.25 ^abc^	0.19 ^ab^	0.32 ^c^	0.15 ^a^	0.18 ^ab^	0.02	<0.001
**Aldehydes**														
2-Methylbutanal	910	43	2.14 ^ef^	1.74 ^abcd^	1.60 ^abc^	1.43 ^a^	1.56 ^ab^	2.26 ^f^	2.04 ^def^	2.70 ^g^	1.95 ^cdef^	1.81 ^bcde^	0.08	<0.001
Hexanal	1089	43	1.47 ^abc^	0.68 ^a^	0.54 ^a^	4.35 ^d^	2.47 ^bc^	2.64 ^bc^	1.12 ^ab^	2.98 ^cd^	0.46 ^a^	0.73 ^a^	0.34	<0.001
Heptanal	1193	57	0.08	0.07	0.07	0.09	0.09	0.11	0.09	0.11	0.09	0.09	0.01	0.050
Octanal	1295	57	0.12 ^a^	0.12 ^a^	0.11 ^a^	0.32 ^c^	0.25 ^bc^	0.29 ^c^	0.17 ^ab^	0.32 ^c^	0.08 ^a^	0.13 ^a^	0.02	<0.001
Nonanal	1404	57	0.92 ^a^	0.82 ^a^	0.86 ^a^	2.15 ^d^	1.76 ^bcd^	1.95 ^cd^	1.25 ^abc^	2.41 ^d^	0.89 ^a^	1.04 ^ab^	0.18	<0.001
**Ketones**														
Acetone	826	70	33.93 ^cd^	31.82 ^cd^	29.65 ^bcd^	21.78 ^ab^	17.96 ^a^	45.88 ^e^	37.68 ^cde^	39.32 ^de^	28.31 ^bc^	32.92 ^cd^	2.16	<0.001
2-Butanone	911	72	0.56 ^de^	0.46 ^abcd^	0.39 ^abc^	0.34 ^a^	0.37 ^ab^	0.61 ^ef^	0.55 ^de^	0.71 ^f^	0.54 ^cde^	0.50 ^bcde^	0.03	<0.001
2-Pentanone	987	43	2.31 ^d^	1.22 ^ab^	1.18 ^a^	2.11 ^cd^	2.20 ^cd^	2.30 ^d^	2.53 ^d^	1.92 ^bcd^	1.24 ^ab^	1.56 ^abc^	0.16	<0.001
2-Heptanone	1187	43	2.10 ^ef^	1.73 ^abcd^	1.61 ^abc^	1.43 ^a^	1.56 ^ab^	2.26 ^f^	2.04 ^def^	2.70 ^g^	1.95 ^cdef^	1.84 ^bcde^	0.08	<0.001
3-Octanone	1260	42	0.03	0.03	0.03	0.03	0.03	0.03	0.03	0.03	0.03	0.03	<0.01	0.653
Acetoin	1304	45	2.10 ^ab^	2.29 ^ab^	2.54 ^abc^	7.55 ^bcd^	8.15 ^cd^	6.26 ^abcd^	8.91 ^d^	1.25 ^a^	1.99 ^ab^	2.46 ^abc^	1.29	0.008
Butyrolactone	1680	42	2.45 ^c^	2.99 ^c^	2.22 ^bc^	2.25 ^bc^	2.49 ^c^	2.97 ^c^	2.82 ^c^	4.32 ^d^	1.16 ^a^	1.41 ^ab^	0.23	<0.001
**Hydrocarbons**														
Z-2-Octene	873	43	0.06 ^bc^	0.02 ^ab^	0.02 ^ab^	0.02 ^ab^	0.02 ^ab^	0.01 ^a^	0.02 ^abc^	0.02 ^ab^	0.07 ^c^	0.13 ^d^	0.01	<0.001
Isododecane	953	57	0.70	1.24	2.02	1.04	1.27	1.34	1.91	2.01	1.51	1.28	0.30	0.088
Beta-Pinene	1110	93	0.30 ^a^	0.78 ^ab^	0.94 ^b^	0.57 ^ab^	0.75 ^ab^	0.99 ^b^	1.06 ^b^	1.01 ^b^	0.67 ^ab^	0.60 ^ab^	0.12	<0.001
**Furan**														
2-Ethylfuran	959	81	0.32 ^ab^	0.26 ^ab^	1.31 ^e^	1.06 ^cde^	0.66 ^abcd^	0.78 ^bcde^	0.53 ^abc^	1.23 ^de^	0.14 ^a^	0.35 ^ab^	0.13	<0.001
**Sulphur compounds**														
Carbon disulphide	745	76	17.04	16.73	14.54	15.93	21.03	12.98	13.10	14.72	15.33	20.52	4.09	0.987
Dimethyl sulphide	959	62	0.77 ^ab^	0.93 ^ab^	1.14 ^bc^	1.44 ^bcd^	2.02 ^d^	1.79 ^cd^	1.92 ^cd^	2.20 ^d^	0.18 ^a^	0.14 ^a^	0.19	<0.001
Dimethyl sulfone	1939	79	7.33 ^bcde^	4.48 ^ab^	6.30 ^abcd^	4.84 ^abc^	3.68 ^a^	12.44 ^f^	10.11 ^ef^	6.11 ^abcd^	8.00 ^cde^	8.14 ^de^	0.72	<0.001

^a, b, c, d, e, f^ Different letters within the same row denote significant difference between means (*p* < 0.05). ^1^ Calculated RI: calculated linear retention index relative to a series of alkanes C7–C26. ^2^ SEM: standard error of the means.
